# Management of Rett Syndrome in the Controlled Multisensory (Snoezelen) Environment. A Review with Three Case Stories

**DOI:** 10.1100/tsw.2006.159

**Published:** 2006-07-08

**Authors:** Meir Lotan

**Affiliations:** Israeli Rett Center, National Evaluation Team, Chaim Sheba Medical Center, Tel HaShomer, Ramat Gan; Zvi Quittman Residential Centers, Israel Elwyn, Jerusalem; Department of Physical Therapy, Academic College of Judea and Samaria, Ariel; and Office of the Medical Director, Division for Mental Retardation, Ministry of Social Affairs, Jerusalem, Israel

**Keywords:** Rett syndrome, Snoezelen, multisensory stimulation, Israel

## Abstract

Rett syndrome (RS) is a neurological disorder resulting from an X-linked dominant mutation. It is characterized by a variety of physical and perceptual disabilities, resulting in a need for continuous intervention programs to be administered on a regular basis throughout life. Many of these individuals with RS show fear of movement and, therefore, find it hard to accept external facilitation (so common in physical therapy intervention). In a search for novel intervention techniques that might improve their ability to cope with difficulties in daily situations, while also reducing their difficulty in handling motion inflicted by an external physical facilitator, we examined the use of the Snoezelen room. The Snoezelen, also known as the controlled multisensory environment, can provide a soothing atmosphere that appeals to the individual with RS, while at the same time it can improve physical, sensorial, and functional abilities. This article suggests various intervention goals that are appropriate for individuals with RS at different stages of the disorder. Since the management of young children with RS in the multisensory environment has been discussed at length in the past, this article will mainly describe intervention with adults with RS, focusing on three case stories. The present article reviews the available scientific materials on the topic of Snoezelen, incorporating clinical knowledge in the field of RS and suggesting this approach as an appropriate intervention method for this population.

## INTRODUCTION

Rett syndrome (RS) is a neurological disorder resulting from an X-linked dominant mutation[1] that affects mainly females and is found in a variety of racial and ethnic groups worldwide[2]. RS is a frequent cause of neurological dysfunction in females, accounting for the second most common cause for multiple-disability among females, after Down's syndrome[3,4].

Individuals with this disorder show a variety of difficulties in motor planning and execution (apraxia) in function, in communication, as well as an array of orthopedic and neurological problems[5]. Since life expectancy of RS patients is estimated to be 49–50 years of age[6], and due to the vast physical challenges experienced by children with this disorder, treatment (especially physical treatment) is essential, necessitating lifelong therapeutic intervention[7]. The secondary motor deterioration typical of adolescents with this disorder compels the increasing use of such physical therapy intervention[8] and presents it as one of the most important treatments for individuals with RS, which is applied to almost 80% of girls with this disorder[7].

In spite of its importance, physical therapy can be unpleasant and, at times, even invasive. Such elements of the treatment may induce unpleasant feelings that the person cannot express. Individuals with RS function according to their highly developed emotional state[9], and when they dislike physical treatment, they might react by resisting, even to the point of falling asleep or evoking a seizure[10]. Due to sensorial difficulties, such as fear of movement, some individuals with RS take little pleasure in external facilitation and, in many cases, show resistance and fear of physical intervention[11]. Due to the above-mentioned consideration, a novel intervention environment was considered. One such approach that could integrate the need for movement and sensorial deficits of the individual with RS, while at the same time present a calmer/emotional experience, is the Snoezelen or controlled multisensory environment (CMSE).

## Snoezelen/Controlled Multisensory Environment

The word Snoezelen is a combination of two Dutch words: Snoezel = to sniff and Doezelen = to doze. The word "doze" indicates that a restful activity is involved and the word "Sniff" gives it the more dynamic sensorial aspect[12]. Ad Verheul, the Dutch inventor of this technique, mentions that the name "Snoezelen" came up due to its nice ring, and therefore clients caught on to the name very easily[12].

The multisensory room is a partially lit room that provides sensory stimulation to both the client and the therapist, where the senses are provided harmoniously whether alone or combined. The sensory stimulus in Snoezelen is provided according to the client's choice, thus empowering the individual participating in such a program. The aim of the treatment is to find a balance between relaxation and activity within the framework of a safe, adapted environment by means of an enabling therapist[13].

We live our lives through our senses. It is by means of experiencing those senses that we develop an understanding about our environment[14]. Any form of sensorial disturbance that a person may exhibit, which will disturb her interaction with the environment, may influence her understanding and, as a result, may disturb development. According to Longhorn[15], without sensation and arousal of the senses, people with disturbances will find it very difficult to comprehend the world around them and, as a result, will have difficulty learning.

The basic assumption is that the CMSE intrigues the client's interest, therefore, she begins to explore and discover her surroundings. This exploration acts as a stepping stone towards learning.

People with disabilities usually have minimal control over their own lives and cannot create their own optimal environment. The Snoezelen gives them the opportunity to do so. By building the CMSE according to the client's preference, we create a safe and favored sensory experience for the individual with RS to explore[13].

Intervention in the Snoezelen with different populations with disabilities was found to be highly effective in addressing various problems[16]. Holtkamp and Kragt[in 17] undertook a study where Snoezelen was applied with 17 elderly persons. Using a randomized cross-over design, they indicated a higher degree of well-being for the experimental group than for the control group. Shapiro et al.[18] conducted an experimental cross-over design study with 20 children with intellectual disability (not including children with RS) exhibiting maladaptive behaviors. This research concluded that Snoezelen had a positive effect as a therapeutic agent on these children. The research included both behavioral and physiological measures. The results of their ambulatory heart rates suggested that the Snoezelen could balance heart rate, reducing it in children who were hyperactive and increasing it in children who were passive[18]. Snoezelen was also found to be effective in dealing with individuals with apraxia[19], to yield positive physical outcomes, such as improving gate and balance for individuals with physical disabilities[20], and to show carry-over effects, generalizing into daily situations, for individuals presenting extreme agitation and challenging behaviors[21,22]. These indirect findings, as well as the author's experience[23,24], imply that this environment might be adjusted for the individuals with RS at various ages and with a variety of difficulties.

## Implementing the Multisensory Environment for Individuals with RS

It should be iterated that every person with RS is unique and that the individual clinical expression of this disorder necessitates a thorough evaluation for each person prior to deciding on the type and content of intervention delivered, preferably in a multidisciplinary approach[25]. Despite such individual diversity, those with RS display some typical characteristics that might be appropriate to handle through treatments via the multisensory environment. Common challenges of this population should be screened according to the typical stages of the disorder.

When screening the literature, one might find hints as to intervention elements that experts in the field of RS have suggested as beneficial for this population:

- Close interpersonal contact[26] is recommended.
- Quiet and reassuring environment may achieve reduction in anxiety and agitation in individuals with RS[27].
- Music is valuable as means of relaxation[28].
- The individual (with RS) will respond best to tender loving care, which encourages activity in an interesting, but quiet, environment during regression[28].

In a lecture by Kerr[29], the following suggestions for a desired intervention with this population were added to the above:

- Encourage and facilitate learning without pressurizing the child.
- Provide face-to-face contact, talking, singing, touching.
- Comfort the child and allow withdrawal during agitation.
- Encourage active supervised movement in soft play.
- Provide gentle movement of limbs and joints through their full range of motion.

When examining the CMSE and the concept of the enabling therapist, we find that it incorporates all the above elements and can serve as a potential intervention program for the individual with RS. A person with RS will respond positively to face-to-face contact, gentle caressing, and communicative sounds since those responses are programmed from a young age in every child[9]. Besides calming the agitated person, the interactions available in the multisensory environment might encourage the emergence of hidden skills such as reaching out and initiation of hand manipulation, exploration behaviors, and vocalization.

General clinical guidelines when interacting with person with RS are as follows:

- The therapist should always be attuned to nonverbal language expressed by the eyes and face of the client with RS[30], constantly talking to her, explaining what will happen next, while reinforcing her activities.
- About 73% of individuals with RS present some form of epilepsy[29], thus, it is advisable to avoid different visual stimuli that might cause or induce a seizure (i.e., flickering lights).

- The role of the therapist in the multisensory environment is to create an interpersonal atmosphere in which the client feels unconditionally accepted[13].
- Music has constantly been found to be of enormous value for individuals with RS, at all levels of disability and ages[30,31,32,33,34,35]. The right kind of music can facilitate learning, relaxation, or may on the other hand, excite the individual with RS. Advice regarding the recommended music for each individual should be predetermined according to the therapeutic goals and might be obtained from parents, caregivers, and the individual's music therapist.
- The therapist should position him/herself according to the client's preferences and therapeutic needs. The therapist can be facing the individual and communicating while massaging or moving her body and limbs[36], or can be behind the person with RS, safely containing the her with his arms, as described by Kerr and Burford[37].

## Management of Typical Difficulties According to Stages of RS that Might be Addressed in the CMSE

The importance of careful and specific evaluation is reiterated for each client before deciding her needs, difficulties, and personal preferences. Nevertheless, most individuals with RS go through the stages listed below and some may present similar characteristic and disabilities. This next section will touch on such common features.

### *Stage I: ONSET*

The development of most RS females is close to normal, the symptoms of RS are just emerging at this stage and are somewhat vague. At this point, most children will display hypotonia and might even present improved finger usage. In most cases, the girl will usually not alarm even the most competent of physicians. The second stage is where the aberrant development emerges and the child's situation receives ample attention. The next stage can have a rapid onset and is a very difficult period for the girl[36].

### *Stage II: DESTRUCTIVE STAGE/MOTOR DETERIORATION*

Although relatively short (spanning weeks or up to a few months), this stage is the girl's (as well as her family's) most difficult experience. Now she completely lacks orientation, her senses are severely disturbed, and her body betrays her[10].

Getting into the quiet, dim-lighted environment at this stage may ease the child's discomfort. The waterbed might serve as a peaceful "island in the midst of chaos"[36]; it gently embraces the girl and takes away the pain, fear, and uncertainty for the duration of the treatment. The personal touch, the eye contact, and the therapist's well-trained and secure hands are all beneficial. Although it is questionable if functional goals can be achieved at this stage, relaxation is bliss. The therapist should try to bond with the child in preparation for future meetings.

The benefits of Snoezelen for young children with RS have already been discussed at length elsewhere[23] and will only be touched on at this point. At this stage of RS, abnormal neural activity can be detected in brain electrical measurements[38] and in biochemical components such as high levels of glutamate[39], causing the cortex and other brain areas into an abnormally excitatory state[27,40]. The severity of such manifestations may be controlled by external intervention, which regulates incoming sensory input and balances the overexcited neural system. With increasing knowledge of RS within the medical community, the diagnosis of young children with RS is progressing, as can be seen in Fig. 1 (published with permission)[41].

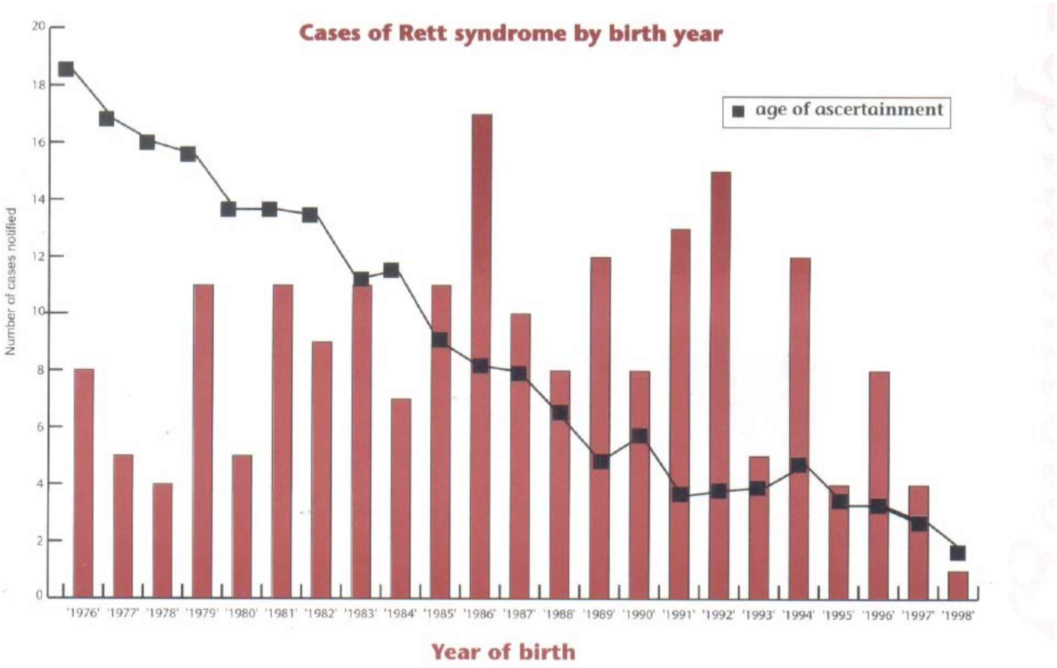

**FIGURE 1. Case of Rett syndrome, ascertainment by birth year.**

It is possible that in the near future, early diagnosis will enable early intervention for children with RS. If therapeutic measures can be utilized at an earlier age, there is a chance for minimizing damage to neural tissue. Experts in the field of neural development[40,42] believe that such (calming) measures, if introduced to the small child with RS, reduce the damage caused by this hyperexited state of the brain tissue, enabling future development and advancement for the child as she enters calmer stages of the disorder (stage III). It is also hypothesized that such a relaxing intervention might have the capacity to normalize the unbalanced sympathetic activity by elevating the basically low-vagal tone typical of RS[31]. The evidence regarding continued brain growth and the absence of major destructive processes[43,44] provide hope that appropriate calming techniques implemented from a young age will enable future brain development and possible future learning[45].

Appropriate intervention goals for individuals at stage II of RS would be **relaxation** techniques. Individuals with RS tend to go through mood swings (although not all of them do and those who do are not always prone to them). At times, we might see that the child is extremely upset without our understanding of the cause of such behavior. Since communication in the majority of girls with RS is nonverbal and, thus, sometimes difficult to understand, it seems that a quiet treatment can save many hours of pain, tears, and discomfort (even if the cause remains enigmatic). It is important to mention that the emotional bonding between the girl and her therapist is extremely important. A loving and understanding therapist will win the girl's love and trust, and as a result, attain her cooperation, essential to effective treatment. With that notion in mind, the therapist should find the time to build an interpersonal relationship. The use of the waterbed with heated water, together with soft, large, pillows/bean bags, can contain the child, enhancing a primal sense of a womb-like experience, which is very calming and is highly recommended. Auditory stimulation in accordance to child's preference will add in building up a sense of familiarity and safeness.

## Case Story One: Relaxation

DA is an adult woman (age, 63 years). DA shows the characteristics of the preserved speech variant of RS. She is independent in eating and walking, but requires help in all other daily activities. DA lives in a residential setting in Jerusalem, Israel. Prior to intervention initiation, when DA was among her peers, she was frequently upset, expressing her unease by excessive body movements and long-lasting, loud shouts (mostly cursing). Since no explanation could be determined to explain her tantrums, it was decided to start her on a Snoezelen intervention program aimed at relaxing her, thus reducing her agitated behavior. DA received two half-hour individual sessions a week. The intervention in the Snoezelen room was performed with dim lights and calming soft music. The attention at the first part of the treatment (about 10 min) was focused on functional abilities and included a tour around the room (see Picture 1) walking (with assistance) on the high mattresses to exercise her equilibrium and walking abilities.

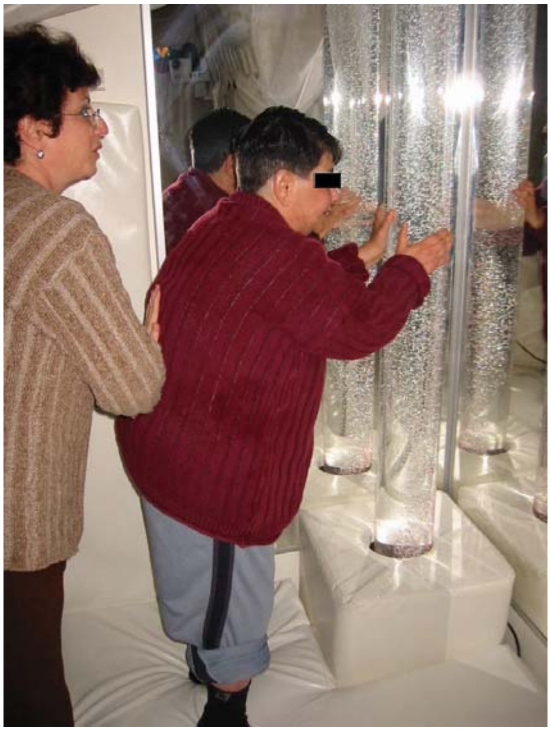

**PICTURE 1.** Touring around the CMSE room

This exercise was presented due to the author’s impression that when stepping on the wide mattresses, there was a change in her pattern of walking from the regular, wide-based, large-sway, straight-kneed gait typical for individuals with RS, to a more normal pattern of walking with bended knees. In the second part of the intervention, DA was lying on her back and receiving a slow and gentle massage to the extremities. This part of the treatment was aimed at reducing her tantrums. DA was enjoying herself during those treatments, came willingly to the Snoezelen room, and was never upset during the intervention (see Picture 2).

After 3 months of intervention, DA's agitation diminished and she was much more relaxed throughout the day (see Fig. 2). Due to the reduction and the complete abolishment of tantrums, the intervention program was terminated in order to enable other clients of the residential setting to benefit from the advantages of the Snoezelen intervention.

## *Stage III: PLATEAU*

This stage is relatively long and calm. The radical physical/emotional situation seems to subside and becomes more balanced. The duration of this stage is quite extended (spanning in terms of years), which enables the girl to achieve some progress due to therapeutic intervention, while preventing possible future problems.

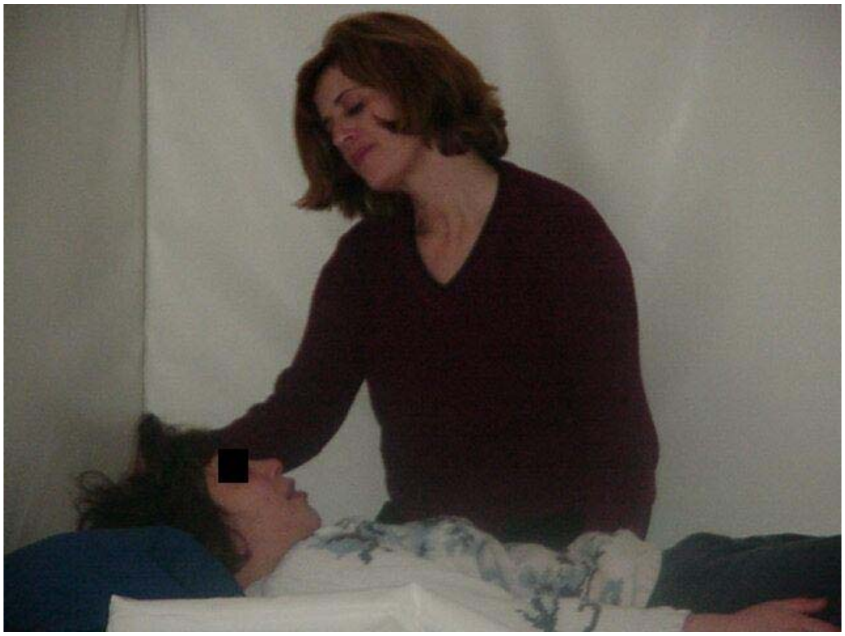

**PICTURE 2.** Achieving relaxation through the CMSE

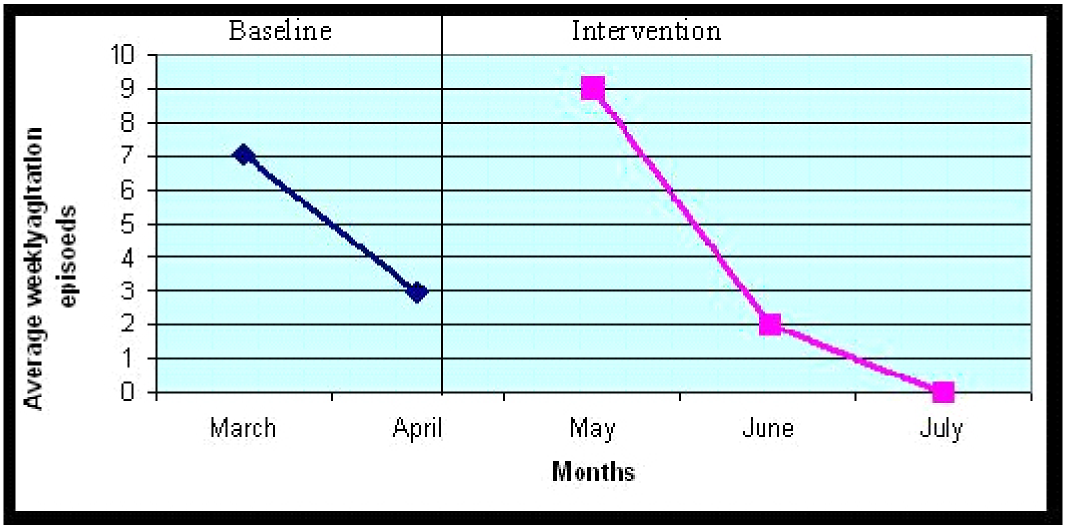

**FIGURE 2.** Case story one (DA): Changes in agitation behavior due to Snoezelen intervention.

Appropriate intervention goals for individuals at stage III of RS would be **hand functions**. Stereotypic hand movements are present in 30–72% of the waking hours of the child with RS[46]. These movements, together with apraxia and ataxia, reduce the likelihood of functional hand use. Thus, in order to activate the hands of the person with RS, it is important to try using various instruments and techniques. An environment that might favorably enhance manual use is the CMSE. Different interesting visual stimuli like “optic fibers”, “infinity”, “fireworks”, and “electrical ball” might be found interesting enough for the client, in order for her to try to use her hands (see Pictures 1 and 3). If the person with RS is able to crawl, performing such an activity on the thick mattresses that cover the floor of the room will facilitate intense proprioceptive stimulation to the hands and might pay off in terms of functional manual improvement. The “bubbles pole” (see Picture 1) creates visual and vibrating stimulation simultaneously and might be used when treatment goals are aimed at enhancing hand function. Different visual stimulation (i.e., through the use of a projector) can be adjusted to illuminate the wall in front of the individual with RS, to further enhance manual function and exploration. The ability to project different slides according to individual client preference can further motivate her to use her hand.

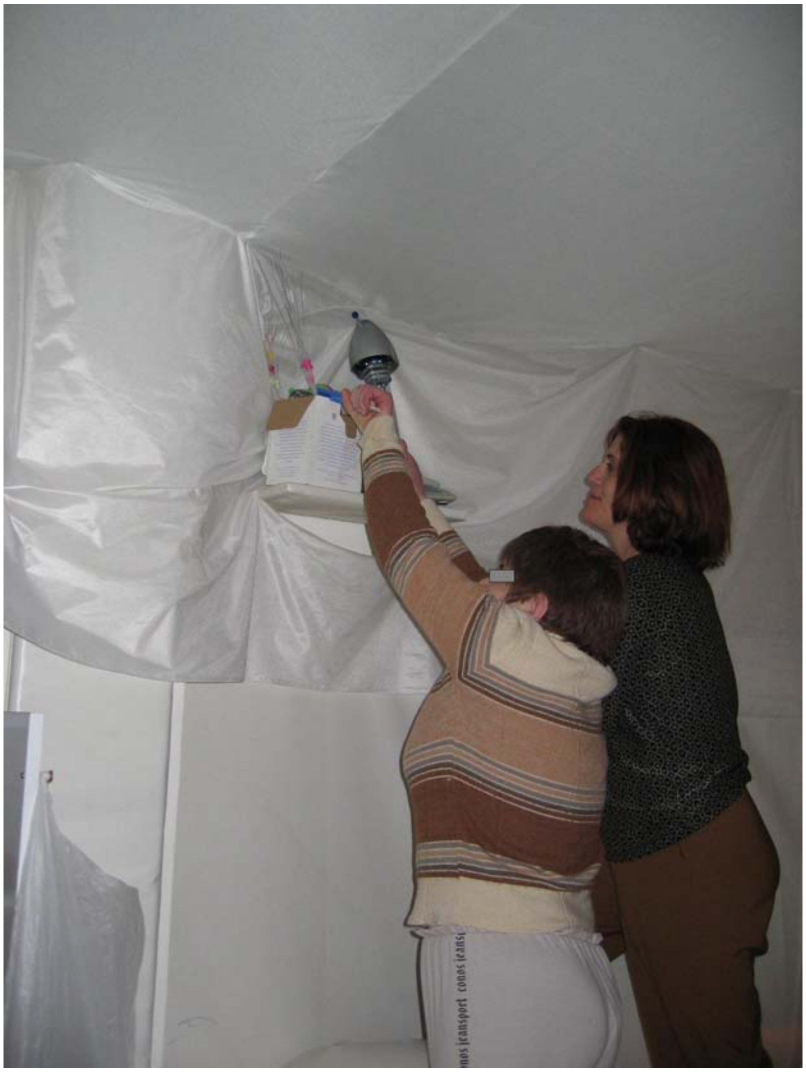

**PICTURE 3.** Achieving functional use through the CMSE.

**Transitions** are another issue at this stage. The primary functional loss for girls with RS is experienced in decreased transitions[47,48]. Transitions include the changes from standing to a sitting position, from sitting to standing, from standing to walking, etc. Therefore, during any functional treatment regime, one should include repetitive elements of movement and position changing on a regular basis[8]. Walking is an extremely important function for individuals with RS because it stimulates the work of internal organs and the digestive system, thus reducing constipation. Standing or walking in an upright position is of immense importance[49] and was found to be positively correlated in delaying the development of scoliosis[50] and reduction of osteoporosis in children with developmental disabilities[51]. The thick, unsteady mattresses create a rough terrain for the individual with RS to handle, but at the same time allows her (as a person usually struggling with the control over her body) to experience walking without the risk of injuring herself in case of a fall. Practicing transitions in the CSME with an adult without RS, but with apraxia, have been found useful in enhancing functional independence and the ability to reperform transitions[19].

**Preventing potential orthopedic problems** (and managing existing ones) should also be a focus at this stage. Girls with RS might display the following orthopedic problems: scoliosis (a side-to-side/rotation curve of the spine), kyphosis (forward curve of the upper thoracic spine), and deformation of the palms and/or feet. Scoliosis can be found in 80–85% of individuals with RS[3,50], especially among nonmobile children[50,52]. Thus, it is very important to provide motivation for walking[49]. Kyphosis is more common among girls who can walk, but is not as severe and motorically disabling as scoliosis[3]. In both cases, the therapist can apply gentle passive mobilization to the trunk, while using the mattresses as a support system. If you manage to get the client with RS to lay on her stomach on the waterbed, it will be extremely beneficial for maintaining and increasing range of motion of the trunk, hips, and shoulder towards extension. The deformation that often occurs in the palms and feet, as well as the dislocation of the hips, require careful attention and specific treatments. The waterbed might be useful in such cases, as well in assisting the therapist as a support system that warms and softens rigid tissues. At the same time, the tranquil and relaxed atmosphere in the multisensory room created by the dim lights, soothing music, and gentle movements of the waterbed can calm the child. This effect will reduce the child's muscle tone and rigidity, and can prevent the disturbing and sometimes painful manual manipulations.

**Reassembling "midline"**[53], as some girls with RS apparently lose their sense of "midline" — In such conditions, the child senses her midline differently from where it actually is. Sometimes they tilt so far to one side or backwards that one might even rule out the possibility of independent seating[8]. This condition prevents her from standing straight (since standing straight seems odd to her) and can cause damage such as scoliosis, kyphosis, and even a loss of ability to sit, stand, and walk. Due to the high frequency of scoliosis in this population[3,50], Hanks[47] suggested that every individual with RS should be routinely treated with deep, symmetrical, proprioceptive stimulation from childhood, as preventive intervention. Rolling on the mattresses is also an option for performing deep, proprioceptive stimulation as part of the multisensory treatment and enables the therapist to work with the individual with RS on her "midline problem" with an electrical massager (if this appliance's sensation can be tolerated by the individual with RS) or without the use of instruments. Covering the child with a big bean-bag sack\pillow can also give intense, proprioceptive input.

**Equilibrium and coordination** — Among her other challenges, the individual with RS is disabled by ataxia. Ataxia is characterized by reduced control over muscle activity in the usual, coordinated manner most of us perform automatically (also displayed by effected postural muscles). The ability to control these muscles is essential for supporting the body in various positions and in movement. Lacking this ability can aggravate motor limitations. Knee-walking across the mattresses of the CMSE can improve equilibrium and trunk control, which is vital for most motor acts. It is postulated that the functional improvement observed after treadmill training in this population was due to enhanced trunk control[54]. Standing on all fours on the waterbed is a challenging exercise and will yield similar results. In addition, such an exercise gives proprioceptive input to the upper extremities and might also enhance hand usage.

## Case Story Two: Improving Gait and Balance in a Woman with RS

BA is an adult woman (age, 58 years) showing the characteristics of RS, living in a residential setting in Jerusalem, Israel. She is self-ambulant, but requires assistance in all other daily activities. She is extremely kyphotic. In the recent months prior to intervention initiation, staff informed the therapeutic department that she had started to show signs of old age manifested in increased kyphosis and falls. After discussing the situation, an intervention program was initiated both at BA's dormitory and in the CMSE (Snoezelen). The aims of the program were to reduce falls and to improve posture. The staff was advised to put BA on her stomach for 20 min every day during nap times. In the Snoezelen, BA received two half-hour-long individual sessions a week. The lights were kept on and popular music was played in the background. During those sessions, BA would walk around the room, stepping on the thick mattresses in order to improve her balance and was assisted in obtaining motivating objects, which were placed on high shelves across the room (the objects were mainly made up of various small musical and illuminating gadgets, which BA particularly likes) in order to enhance active extension of the arms and upper thorax (see Picture 3).

The program has been going on for 2 years now and is still under way today. According to collected data over a period of 6 years, falls that dramatically increased at 2004, which resulted in the Snoezelen intervention, have completely ceased and are no longer a problem for BA (see Fig. 3) although her posture does not seem to visually change.

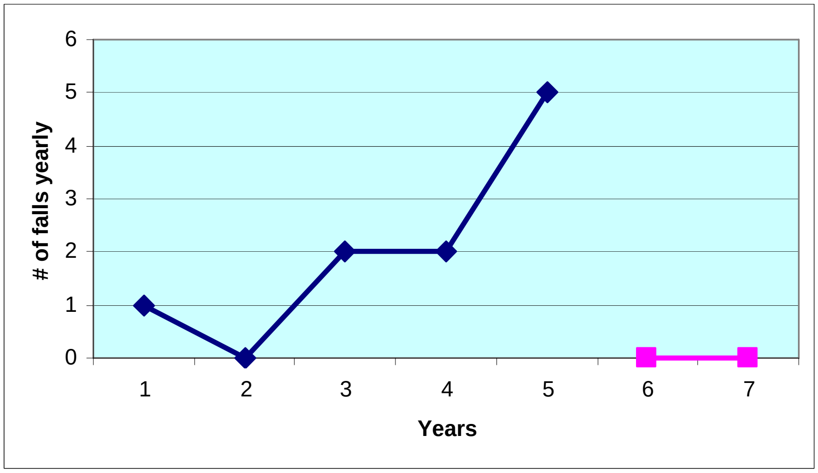

**FIGURE 3.** Improving balance and reducing falls through the use of the CMSE.

**Sensation** — Individuals with RS are often characterized by an unbalanced sensory system. This phenomenon was never thoroughly investigated; nevertheless, it has been well documented mainly by parental reports. Many individuals with RS display decreased pain sensation and hypersensitivity to touch on their faces[55], especially around the mouth[10]. Their fear of movement can be caused by a dysfunction of the vestibular system[10]. As a consequence of her fear of movement, the individual is cautious and the fluency of her everyday life is interrupted. The unclear/disorienting messages received at the cortex level causes the brain to create a distracted body scheme; a vital component in accepting and relating to the world. Without it, the individual is affected by apraxia[14]. The fright of movement can also be express by "gravitational insecurity" presented by a fear of external movement facilitation and ambulation over altered surfaces. Embracing the child should provide her with deep, proprioceptive sensory stability and this secure sensation should be maintained as the basic "sanctuary" until the child is acclimated with the environment, the therapist, and the intervention program. In extreme cases of fear of movement, this relaxed and familiar posture should be the point, where slow rhythmic movement starts from and returns to. After a sense of security in this familiar situation is achieved, the girl who is generally afraid of movement might agree to more physically powerful, free-flowing movements. When the child is completely confident in this situation (in other words, most parts of her nervous system are relaxed and learning can become possible), the sensory feedback enables an integration and organization of her sensory system[14]. Routine visitations to the CMSE to re-establish the confidence in movement might ensure positive results in her daily activities. From my experience, individuals with RS who have shown an obvious and paralyzing rejection towards movement by an external facilitator prior to the intervention and handled in the above-mentioned manner in the CMSE, were gradually accustomed to movement and have opened up to the experience of motion.

## *Stage IV: LATE MOTOR DETERIORATION*

At this stage, which occurs in some girls and women, late motor deterioration may be noticed that requires increased physiotherapy. Several cases have been reported, where girls with RS lost their ability to walk, but were able to improve their functional mobility and regain ambulation[48,56,57]. In one case study, a rehabilitation of lost walking skill was achieved with a 43-year-old individual after 2 decades of immobility[58]. It is important to stress that the inability to walk increases the risk of secondary complications, such as scoliosis, pelvic deformities, pulmonary (lung) problems, and reduced bowel movements and blood flow to internal organs and lower limbs. Thus, it is extremely important to keep individuals with RS ambulant.

The need for extensive intervention collides with her aversion to physical intervention, while the CMSE, with its relaxing effect, can help the child to like or at least endure the obligatory physical manipulations. Despite new difficulties, new areas are open to improvement, such as a decrease in repetitive hand movements and the improvement of social and communicative skills. In stage IV, therefore, one should address the problems reported in stage III, if appropriate and even more often (if necessary), but without neglecting the new areas (improvements).

**Communication**[59] — Most girls with RS do not communicate verbally. However, they like to communicate with others, especially adults, and it is easier for them to communicate in a one-on-one situation (individual treatment). During therapy in the multisensory environment, if communication enhancement is to be achieved, the therapist should be in front of the girl, facing her and maintaining direct eye contact with her. The therapist should be attuned to nonverbal cues expressed by the eyes and face, constantly talking to her, explaining what will happen next, and reinforcing her by commenting on her activities and communicative acts. Moving across the multisensory environment can be performed according to child’s preference using a set of plastic cards (showing different areas of the room) pointed out by the child’s eye gaze or hand (Picture 4). Such activity might have a carry-over to daily situations[60].

**Spasticity and range of motion** (see case story 3) — At an older age, individuals with RS tend to develop high muscle tone. Reducing this spasticity/rigidity requires a relaxed and secure atmosphere, deep manual pressure, and slow, continuous rhythmic movements that can be achieved using the waterbed. The music bed can be used for the same purpose, since the vibration of the loudspeakers can induce muscle tone reduction. Muscle tone reduction has been found useful, especially when using Vibro-acoustics[35].

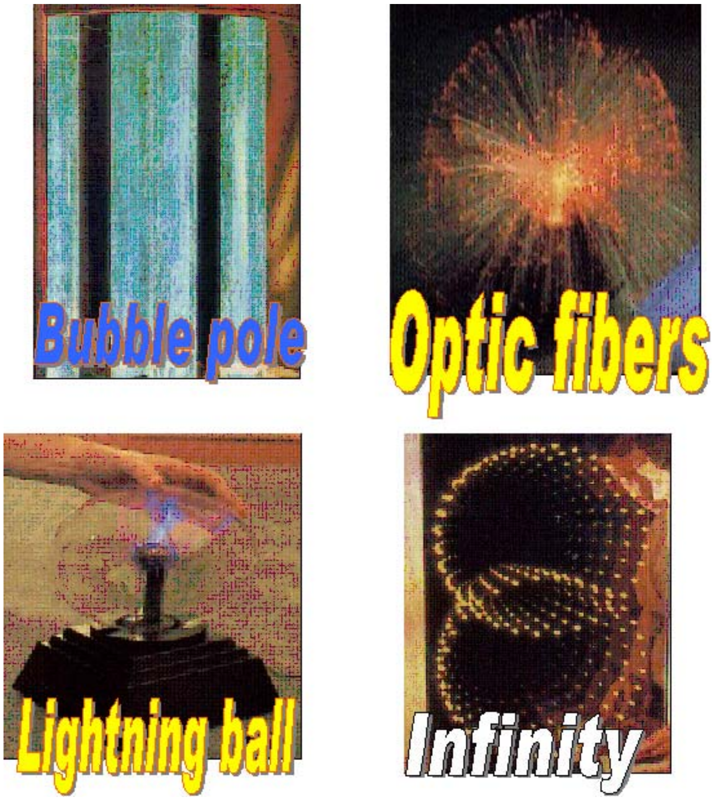

**PICTURE 4**. Enhancing communication through choice-making cards in the Snoezelen environment.

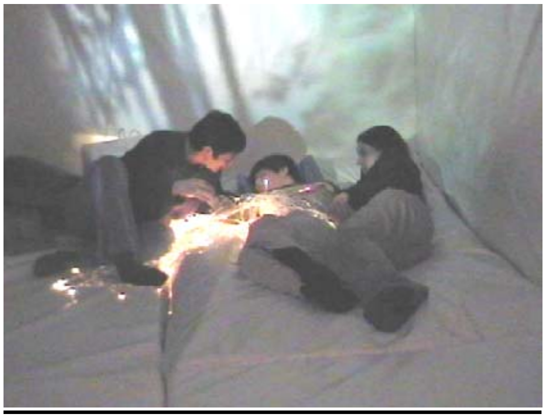

**PICTURE 5**. Familial togetherness in the Snoezelen.

## Case Story Three: Improving Articular Range of Motion

MB is a 24-year-old female diagnosed with RS and living in a residential facility. MB had never walked, had no functional abilities, and showed severe musculoskeletal contractures, both in the trunk and limbs. In recent years, her contractures had deteriorated to the point that her caregivers started to complain that even dressing and undressing her had become a very tedious task, not to mention the pain these everyday occurrences seemed to cause. The constant fear of adding the suffering of a pathological fracture due to the fragility of RS bones[61,62,63] was unremittingly present.

In light of this situation, a program for increasing her articular range of motion was initiated in the physical therapy room. Despite the fact that this intervention program took into account her long-lasting condition and her fragility, and was delivered in the gentlest manner, it became apparent after the first few sessions that MB was not enjoying the intervention. On top of that, only minimal movements could be performed on her limbs and trunk, which made it clear that this form of intervention was not very efficient and that new intervention options should be considered.

The next option to consider was the Snoezelen environment. MB was introduced to the Snoezelen environment in a slowly adaptive manner and started receiving manual intervention aimed at increasing range of motion in two half-hour sessions per week. The treatment took place on the heated waterbed and was performed with dim lights and soft music. At the beginning of each treatment (5–10 min), MB's body was covered with a large bean bag in order to enhance a feeling of security and supply proprioceptive sensory input, thus reducing muscle tone and relaxing her body. During the remaining part of the intervention, range of movement exercises were performed (in the same manner as it was done before in the physical therapy room) while the slow rhythmic movements, dim lights, and soft music remained constant. Judging by the look in her eyes and the type of smiles she wore, it was obvious that MB was enjoying the experience.

An examination of her joint range of motion (JROM) after the course of 4 months (see Table 1) revealed that an increase in JROM was established throughout her body, enough to ease the daily suffering of MB and alleviate her caregivers' difficulties. Moreover, this small change has been found statistically significant.

## CONCLUSIONS

It is reiterated that the present article is a review of the available scientific materials on the topic of Snoezelen, incorporating the clinical knowledge of the author in the field of Rett syndrome; therefore, scientific generalization should be limited. This article and other available literature on this topic[23,24] illustrated the possibility of using the controlled multisensory environment (CMSE/Snoezelen) as an appropriate intervention approach for young girls as well as adult women with RS towards achieving a variety of remedial goals. The fact that the multisensory environment enabled the use of the two major motivational factors of individuals with RS (love for music and close human contact) is by itself a good reason for suggesting such a program for clients with RS. Moreover, it appears that when screening some of the major difficulties of individuals with RS, most of them might be cared for using this approach. Nevertheless, it is reiterated that any management program should be adapted to the individual client after a careful evaluation of her specific needs.

When evaluating the child with RS, it is also important to realize the possible future achievements and plan ahead, while deciding on new ways of intervention with those individuals[64]. Since individuals with RS tend to have an overactive sympathetic system[65] that constantly places them in a state of highly strained being, using a soothing environment such as the Snoezelen on regular basis might contribute to relaxation and as a result, to an enhanced quality of life. An additional important element to be reflected on, when considering the Snoezelen room as a therapeutic approach for individuals with RS, is our ability to change the setting according to each client's preferences, thus empowering her. It should be emphasized here that the use of Snoezelen as described in the present article adds to the original concept of the Snoezelen by using this approach in a more therapeutic manner than intended by the original initiators of this approach[12]. This manner of operating intervention in the CMSE is coined by the author as Therapeutic Snoezelen Intervention (TSI), which gives an added value for this environment as long as the therapeutic endeavors do not contradict with the basic concept of the enabling therapist.

**TABLE 1**
**Difference in Range of Motion after a 4-Month Intervention**

| Body Part | Side | Before, 15/6/2004 | After, 13/10/2004 | Direction of Movement |
|---|---|---|---|---|
| Upper Extremities | | | | |
| Shoulder | Left | 65 | 92 | Flexion |
| | | 19 | 180 | Extension |
| | | 90 | 113 | Elevation |
| | | — | 45 | Internal rotation |
| | | — | 65 | External rotation |
| | Right | 75 | 180 | Flexion |
| | | 180 | 180 | Extension |
| | | 62 | 90 | Elevation |
| | | — | 180 | Internal rotation |
| | | — | 180 | External rotation |
| Elbow | Left | 180 | 180 | Flexion |
| | | 100 | 113 | Extension |
| | Right | 180 | 180 | Flexion |
| | | 107 | 105 | Extension |
| Palm | Left | 80 | 80 | Flexion |
| | | 10 | 22 | Extension |
| | | 45 | 45 | Ulnar dev. |
| | | –10 | 0 | Radial dev. |
| | Right | 0 | 20 | Flexion |
| | | 90 | 90 | Extension |
| | | 45 | 45 | Ulnar dev. |
| | | –10 | 0 | Radial dev. |
| M.C.P | Left | 110 | 110 | Flexion |
| | | 10 | — | Extension |
| | Right | 32 | — | Flexion |
| | | 0 | — | Extension |
| Fingers | Left | 180 | 180 | Flexion |
| | | 0 | 0 | Extension |
| | Right | 180 | 180 | Flexion |
| | | 0 | 0 | Extension |
| Lower Extremities | | | | |
| Hip | Left | 95 | 180 | Flexion |
| | | –15 | 0 | Extension |
| | | 0 | 0 | Abduction |
| | | 0 | 0 | Adduction |
| | | 90 | 90 | Internal rotation |
| | | 0 | 37 | External rotation |

**TABLE 1 (continued)**

| | | | | |
|---|---|---|---|---|
| | Right | 40 | 180 | Flexion |
| | | 0 | 0 | Extension |
| | | 10 | 30 | Abduction |
| | | 10 | 12 | Adduction |
| | | 0 | 30 | Internal rotation |
| | | 60 | 64 | External rotation |
| Knee | Left | 180 | 180 | Flexion |
| | | 103 | 153 | Extension |
| | Right | 150 | 155 | Flexion |
| | | 60 | 118 | Extension |
| Ankle | Left | 32 | 25 | Dorsi flexion |
| | | 10 | 50 | Plantar flexion |
| | Right | 5 | 10 | Dorsi flexion |
| | | 40 | 40 | Plantar flexion |

A one-way paired student t-test found a significant difference, $p < 0.00033$, between the before and after measurements.

## ACKNOWLEDGMENTS

The author would like to thank the management of Israel Elwyn and the Zvi Quittman residential centers for their cooperation and support in this project.

## BIOSKETCH

**Meir Lotan, MScPT,** is a physiotherapist working on the Israeli National Rett Syndrome evaluation team. He has a special interest in physiotherapy and persons with intellectual disability, Snoezelen, and physical activity for children and adults with intellectual disability with an emphasis on individuals with Rett syndrome. He lectures on assistive technology at the Department of Physical Therapy, Academic College of Judea and Samaria in Ariel and the Ben Gurion University of the Negev in Beer-Sheva. Awarded in 2000 by the IRSA (International Rett Syndrome Association) for his donation to individuals with Rett syndrome. E-mail: ml_pt_rs@netvision.net.il
